# Associations between cardiovascular risk factors and diseases with aortic pulse wave velocity and aortic distensibility: magnetic resonance imaging in the Hamburg city health study

**DOI:** 10.1007/s00392-026-02866-x

**Published:** 2026-02-09

**Authors:** Katharina A. Riedl, Eleonora Di Carluccio, Markus Huellebrand, Anja Hennemuth, Maike Frye, Paula Kaufmann, Mariam Hazizi, Ersin Cavus, Jan N. Albrecht, Enver Tahir, Jennifer Erley, Martin Sinn, Bjoern P. Schoennagel, Gerhard Adam, Paulus Kirchhof, Stefan Blankenberg, Gunnar Lund, Andreas Ziegler, Kai Muellerleile

**Affiliations:** 1 Department of Cardiology, University Heart and Vascular Center, University Medical Center Hamburg-Eppendorf, Martinistraße 52, Hamburg, 20246 Germany; 2 German Center for Cardiovascular Research (DZHK), Partner Site Hamburg/Kiel/Lübeck, Hamburg, Germany; 3 Cardio-CARE, Medizincampus Davos, Davos, Switzerland; 4 Deutsches Herzzentrum Der Charité (DHZC), Institute of Computer-Assisted Cardiovascular Medicine, Berlin, Germany; 5 Charité-Universitätsmedizin Berlin, corporate member of, Freie Universität Berlin and Humboldt-Universität Zu Berlin , Berlin, Germany; 6 Fraunhofer Institute for Digital Medicine MEVIS, Berlin, Germany; 7 German Center for Cardiovascular Research (DZHK), Partner Site Berlin, Berlin, Germany; 8 Department of Diagnostic and Interventional Radiology and Nuclear Medicine, University Medical Center Hamburg-Eppendorf, Hamburg, Germany; 9 Institute of Clinical Chemistry and Laboratory Medicine, University Medical Center Hamburg-Eppendorf, Hamburg, Germany; 10 Institute of Cardiovascular Sciences, University of Birmingham, Birmingham, UK; 11 Centre for Population Health Innovation (POINT), University Heart and Vascular Center Hamburg, University Medical Center Hamburg-Eppendorf, Hamburg, Germany; 12 School of Mathematics, Statistics and Computer Science, University of KwaZulu-Natal, Pietermaritzburg, South Africa

**Keywords:** Pulse wave velocity, Aortic distensibility, Aortic stiffness, Cardiovascular magnetic resonance imaging, Prevention

## Abstract

**Background:**

The role of cardiovascular magnetic resonance (CMR)-imaging-based pulse wave velocity (PWV) and aortic distensibility (AD) in population-based cohorts as a risk stratification tool remains unclear. The purpose of this study was the CMR-based quantification of PWV and AD in the context of cardiovascular risk factors (CVRF) and/or diseases (CVD) in the Hamburg City Health Study (HCHS).

**Methods:**

The HCHS is a prospective, population-based cohort study. 2D-phase-contrast-flow CMR measurements were performed to quantify PWV and AD in the ascending (AD AoAsc) and descending aorta (AD AoDesc).

**Results:**

The CMR cohort consisted of 2270 participants (41.5% females, median age 66.5 years). PWV was 5.80 [4.91, 7.19] m/s, AD AoAsc 0.54 [0.34, 0.78] [1/(10^3*kPa)], and AD AoDesc 0.61 [0.39, 0.84] [1/(10^3*kPa)] in participants without any CVRF and/or CVD. In participants with at least one CVRF and/or CVD PWV was significantly higher, AD AoAsc and AD AoDesc significantly lower. After adjustment for age and sex, PWV was significantly associated with smoking (OR 1.05), CAD (OR 0.932), and hypertension (OR 1.118); AD AoAsc with hypertension (OR 0.448); and AD AoDesc with hypertension (OR 0.343), BMI > 30 kg/m^2^ (OR 0.575), CAD (OR 2.17), and history of myocardial infarction (OR 2.413).

**Conclusions:**

The presence of CVRF and/or CVD is related to significantly higher PWV and lower AD values. However, hypertension is the only CVRF/CVD consistently associated with higher PWV and lower AD after adjustment for age and sex. Our findings do not indicate a predictive value of abnormal PWV and AD values for prevalent CAD and MI.

**Graphical Abstract:**

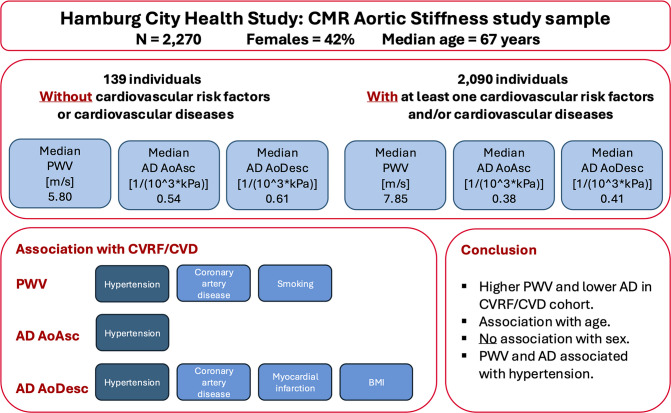

**Supplementary Information:**

The online version contains supplementary material available at 10.1007/s00392-026-02866-x.

## Introduction

Aortic stiffness (AS) is a contributor and a consequence of atherosclerosis and vascular aging [1]. AS can be assessed non-invasively by aortic pulse wave velocity (PWV) and aortic distensibility (AD) [2]. PWV is defined as the ratio of the distance between two measurement points of the aorta and the temporal delay of the pulse wave between these points [3], whereas AD is defined as the relative change of the aortic diameter or area divided by the pulse pressure [4, 5]. PWV and AD correlate inversely since an “elastic” aorta exhibits low PWV and high AD values [6, 7]. Recent studies showed that PWV values correlate with blood pressure and aortic diameters [6]. Moreover, PWV is currently discussed as a predictor of future cardiovascular events and mortality [8]. Therefore, PWV and AD are attractive parameters to identify different cardiovascular phenotypes in response to cardiovascular risk factors and have the potential to facilitate a personalized management of individuals at increased cardiovascular risk [9].

Several methods have been used to quantify PWV and AD: The standard method to quantify PWV non-invasively is the carotid-femoral (cf) method, which is based on applanation tonometry [10]. This method does not consider the ascending aorta and the aortic arch and does not include exact measurements of the distance between the carotid and femoral measurement points [11]. Another method to quantify AS is the calculation of the photoplethysmography-based aortic stiffness index (ASI), which has been used in the UK Biobank [12]. Cardiovascular magnetic resonance (CMR) enables the direct measurement of the distance between two aortic measurement points for a more accurate calculation of the transit time of the aortic pulse wave, overcoming the major limitation of the cf and the ASI method for PWV assessment [13]. Furthermore, CMR also offers measurements of AD by directly measuring the change of the aortic area and/or diameter over time using operator-selected points [4, 5, 14] or algorithms [15]. However, there are no currently available data on associations of PWV and AD by CMR with cardiovascular risk factors (CVRF) and/or disease (CVD) in large, population-based cohorts. Therefore, the purpose of this study was to assess associations of CMR-derived PWV and AD measurements with CVRF and CVD in the Hamburg City Health Study [16].

## Materials and methods

### Study population and design

**Fig. 1:**
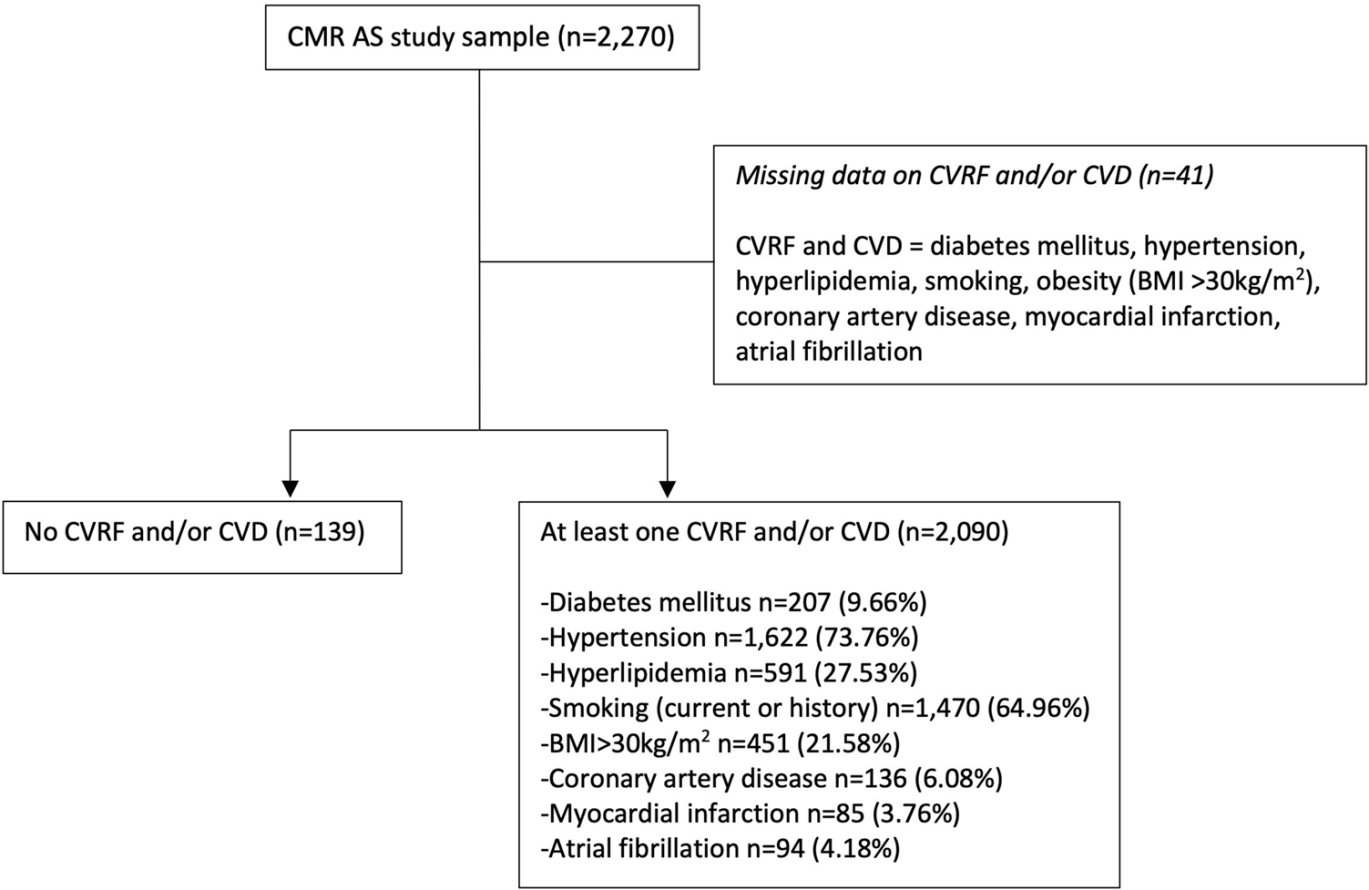
Study population. In 2270 participants of the first 10,000 participants of the Hamburg City Health Study, CMR-based pulse wave velocity and aortic distensibility measurements were available. A total of 2090 participants demonstrated at least one CVRF and/or CVD (CVRF/CVD + group), whereas in 139 participants, no CVRF or CVD (CVRF/CVD − group) was identified. In 41 participants, no information on CVRF/CVD was available. *CMR*,* cardiovascular magnetic resonance*;* AS*,  *aortic stiffness*;* CVRF*,* cardiovascular risk factors*;* CVD*,* cardiovascular diseases*;* BMI*,* body mass index*

The Hamburg City Health Study (HCHS) is a single-center, prospective, long-term, population-based, epidemiological study including participants between 45 and 74 years, who are citizens of the city of Hamburg, Germany. The detailed study design is described and published by Jagodzinski A et al*.* [17]. This analysis is based on the first available 10,000 participants of the HCHS cohort, who were included between February 8, 2016, and November 7, 2018, of whom 2270 participants underwent a CMR scan with PWV and AD measurements **(**Fig. 1**)**. This final CMR AS study sample was divided into a subgroup without any CVRF or CVD (CVRF/CVD − group) and a subgroup with at least one or more CVRF and/or prevalent CVD (CVRF/CVD + group) **(**Fig. 1**)**. CVRF and CVD were defined as current or a history of smoking status, hyperlipidemia, obesity (body mass index (BMI) > 30 kg/m^2^), hypertension, diabetes mellitus, history of myocardial infarction (MI), coronary artery disease (CAD), and/or atrial fibrillation (AF), respectively [16]. The aforementioned CVRF and CVD were self-reported and obtained by standardized questionnaires according to the HCHS study protocol [17].

### Ethics and informed consent

The local ethics committee of the Landesaerztekammer Hamburg (State of Hamburg Chamber of Medical Practitioners, PV5131) did not object to the conduct of the Hamburg City Health Study (HCHS). The study has been registered at ClinicalTrial.gov (NCT03934957) and is conducted in accordance with the Good Epidemiological Practice (GEP) and the Declaration of Helsinki. All participants of HCHS gave their written informed consent.

### CMR protocols

**Fig. 2:**
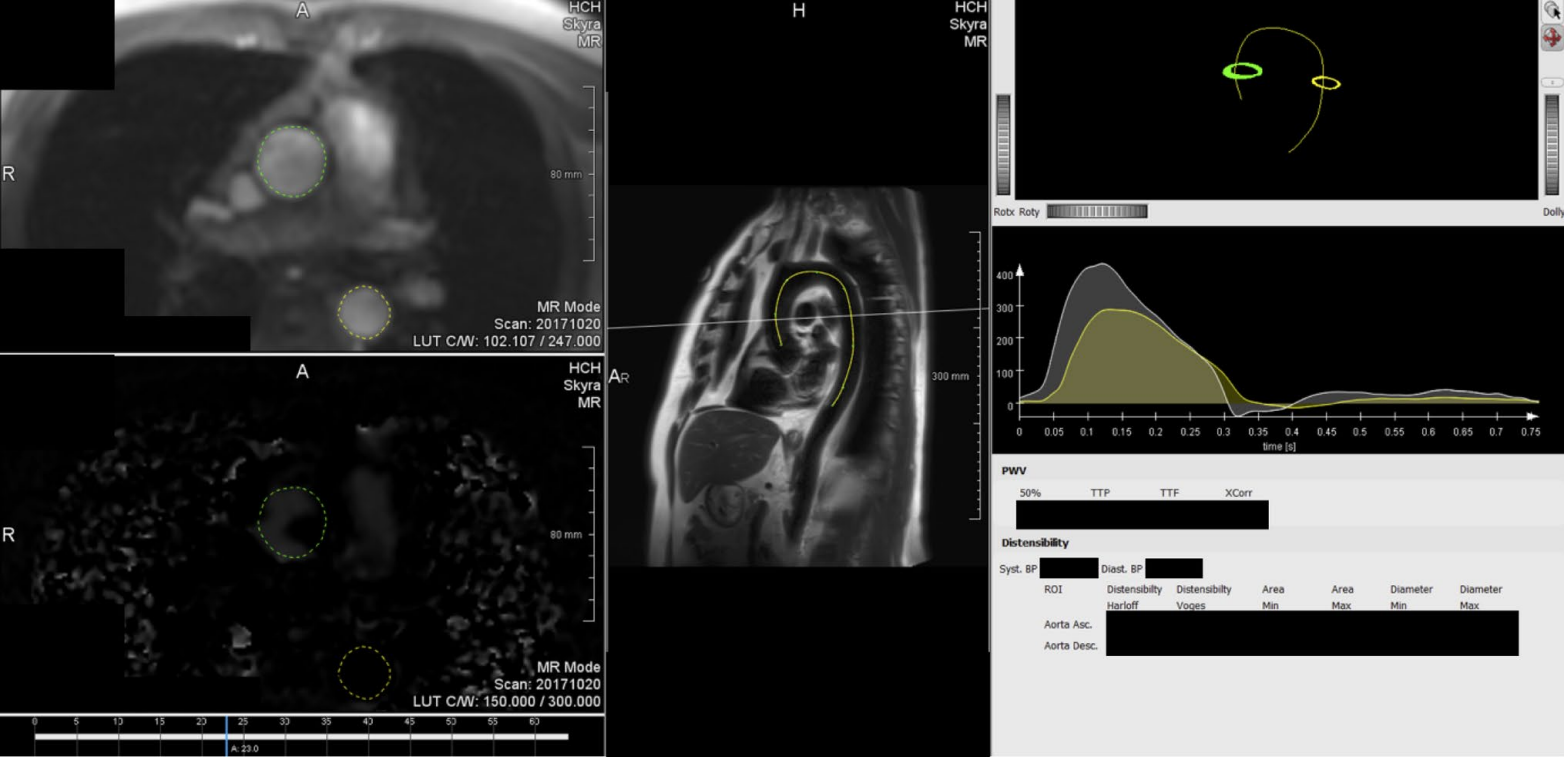
CMR data analysis tool. Pulse wave velocity (PWV) and aortic distensibility (AD) were analyzed using an analysis tool, provided by the working group of Anja Hennemuth (Charité Berlin, Berlin, Germany) [19]. Phase and magnitude slices (left), T2 HASTE scan with aortic centerline (mid), flow curves and PWV, AD and aortic values (right). *CMR*, *cardiovascular magnetic resonance*; *HASTE*, *half Fourier-acquired single shot turbo spin*

All CMR scans were performed at a 3 T magnetic resonance imaging (MRI) scanner (MAGNETOM™ Skyra, Siemens Healthineers, Erlangen, Germany) at the University Medical Center Hamburg-Eppendorf, Hamburg, Germany, under supervision by certified magnetic resonance imaging level III approved radiologists or cardiologists. Standardized CMR scan protocols were used as previously described [16]. In particular, a parasagittal T2 HASTE (Half Fourier-acquired single shot turbo spin) sequence was performed, visualizing the whole aortic arch using the three-point planning technique placing three points in the ascending aorta, the aortic arch, and the descending thoracic aorta in the trans-axial stack set [18]. This approach enabled measurements of the exact distance ($\Delta x$) between the two aortic measurement points in the ascending and descending aorta (Fig. 2). Participants with an incomplete visualization of the aortic arch were excluded from our analysis. Typical T2 HASTE MR parameters were repetition time (TR) 3.8 ms, echo time (TE) 91 ms, flip angle (FA) 110°, voxel size 1.3 × 1.3x8 mm^3^, and field of view (FoV) 400 mm^2^ with a parallel acquisition technique (PAT) factor of 2 [16]. Blood flow in the ascending and descending aorta was measured on a single slice using a 2D phase-contrast velocity-encoding (PC VENC) sequence at the level of the right pulmonary artery (Fig. 2). Typical VENC-CMR parameters were TR 4.3 ms, TE 2.3 ms, FA 20°, voxel size 1.2 × 1.2x8 mm^3^, FoV 300 mm^2^, PAT 2, and 64 acquisitions per heart cycle [16].

### CMR-based PWV and AD analysis

CMR raw data were transferred for PWV and AD analyses to a dedicated, non-commercial analysis tool provided by the working group of Prof. Hennemuth (Charité Berlin, Berlin, Germany) [19] **(**Fig. 2**)**. PWV was defined as the distance between two measurement points, measured by the centerline method on the parasagittal T2 HASTE scan, divided by the transit time of the pulse wave, calculated by placing regions of interest (ROIs) in the ascending and descending aorta [3] **(**Fig. 2**)**. PWV 50% algorithm was used using the time point where the flow rate is half of the peak flow rate [20]. AD was calculated dividing the relative change of the aortic diameter over the heart cycle by the pulse pressure ($\Delta P$) as the difference between the systolic and diastolic blood pressure according to Harloff et al*.* [4]. The aortic diameter was measured by propagating ROIs in the ascending and descending aorta over the entire heart cycle **(**Fig. 2**)**. Pulse pressure was obtained from by non-invasive brachial blood pressure values measured in advance of the CMR scan.

### Statistical analysis

Continuous variables were described as median (25th percentile, 75th percentile) and dichotomous variables as absolute and relative frequencies. Differences between continuous variables were tested by the Mann-Whitney U test in conjunction with a 95% Hodges-Lehmann confidence interval (CI). Association analysis was performed with point-biserial correlations after Box-Cox transformation. Box-Cox transformation was used for the reduction of skewness and approximate normality of continuous variables before estimating point-biserial correlations. We performed age and sex-adjusted logistic regression to investigate the association with cardiovascular risk factors and cardiovascular diseases. PWV and AD were treated as dependent variables. All statistical analyses were done using R 4.3.0. Quarto was used as the markup language to guarantee the reproducibility of all statistical analyses.

## Results

### Study population

A total of 2270 participants underwent a CMR scan for AS quantification. The median age of the final study population was 66.5 [59.0, 72.0] years, and 42% (942/2270) of the participants were female **(**Table 1**)**. CVRF and CVD were absent in 139 (6.12%) participants **(**Fig. 1**)**. A total of 2090 participants had at least one or more CVRF and/or CVD: 74% (*n* = 1622) of the participants had hypertension, 28% (*n* = 591) hyperlipidemia, 10% (*n* = 207) diabetes mellitus, 22% (*n* = 451) an BMI > 30 kg/m^2^, 4% (*n* = 94) atrial fibrillation, 4% (*n* = 85) a history of myocardial infarction, 6% (*n* = 136) coronary artery disease, and 65% (*n* = 1470) were current or history smokers **(**Table 1, Fig. 1**)**. Forty-one participants were excluded and filtered out from this analysis due to missing CVRF and/or CVD data **(**Fig. 1**)**.

**Table 1:** Study population

	CMR AS study sample (*n* = 2270)
Age, years	66.50 [59.00, 72.00]
Sex (female)	942 (41.50)
Systolic blood pressure before CMR (mmHg)	130.0 [120.0, 144.0]
Diastolic blood pressure before CMR (mmHg)	79.00 [73.00, 86.00]
Diabetes mellitus	207 (9.66)
Hypertension	1622 (73.76)
Hyperlipidemia	591 (27.53)
Smoking (current and history)	1470 (64.96)
BMI (kg/m^2^)	26.38 [23.86, 29.27]
Coronary artery disease	136 (6.08)
Myocardial infarction	85 (3.76)
Atrial fibrillation	94 (4.18)
Creatinine (mg/dl)	0.81 [0.74, 0.90]

Continuous data are median and interquartile range. Categorial data are absolute numbers with percentages. *AS*, aortic stiffness; *CMR*, cardiovascular magnetic resonance; *BMI*, body mass index

### PWV and AD in participants with and without CVRF and/or CVD

Median PWV was 5.80 [4.91, 7.19] m/s, median AD AoAsc 0.54 [0.34, 0.78] [1/(10^3*kPa)], and median AD AoDesc 0.61 [0.39, 0.84] [1/(10^3*kPa)] in the CVRF/CVD − group (*n* = 139) **(**Table 2). In the CVRF/CVD + group, median PWV was significantly higher, and median AD AoAsc and AD AoDesc were significantly lower compared to the CVRF/CVD − group (all *p* < 0.001) (Table 2).

**Table 2:** PWV, AD AoAsc, and AD AoDesc according to CVRF and/or CVD

	CVRF/CVD – group (*n* = 139)	CVRF/CVD + group (*n* = 2090)	***p-***values
PWV [m/s]	5.80 [4.91, 7.19]	7.85 [6.33, 9.93]	< 0.001
AD AoAsc [1/(10^3*kPa)]	0.54 [0.34, 0.78]	0.38 [0.25, 0.59]	< 0.001
AD AoDesc [1/(10^3*kPa)]	0.61 [0.39, 0.84]	0.41 [0.27, 0.61]	< 0.001

Continuous data are median and interquartile range. *CVRF*, cardiovascular risk factor; *CVD*, cardiovascular disease; *PWV*, pulse wave velocity; *AD*, aortic distensibility; *AoAsc*, ascending aorta; *AoDesc*, descending aorta

CVRF and CVD were defined as current or a history of smoking, hyperlipidemia, obesity (body mass index > 30 kg/m^2^), hypertension, diabetes mellitus, a history of myocardial infarction, coronary artery disease, and/or atrial fibrillation

### Associations of PWV and AD with age and sex

**Fig. 3:**
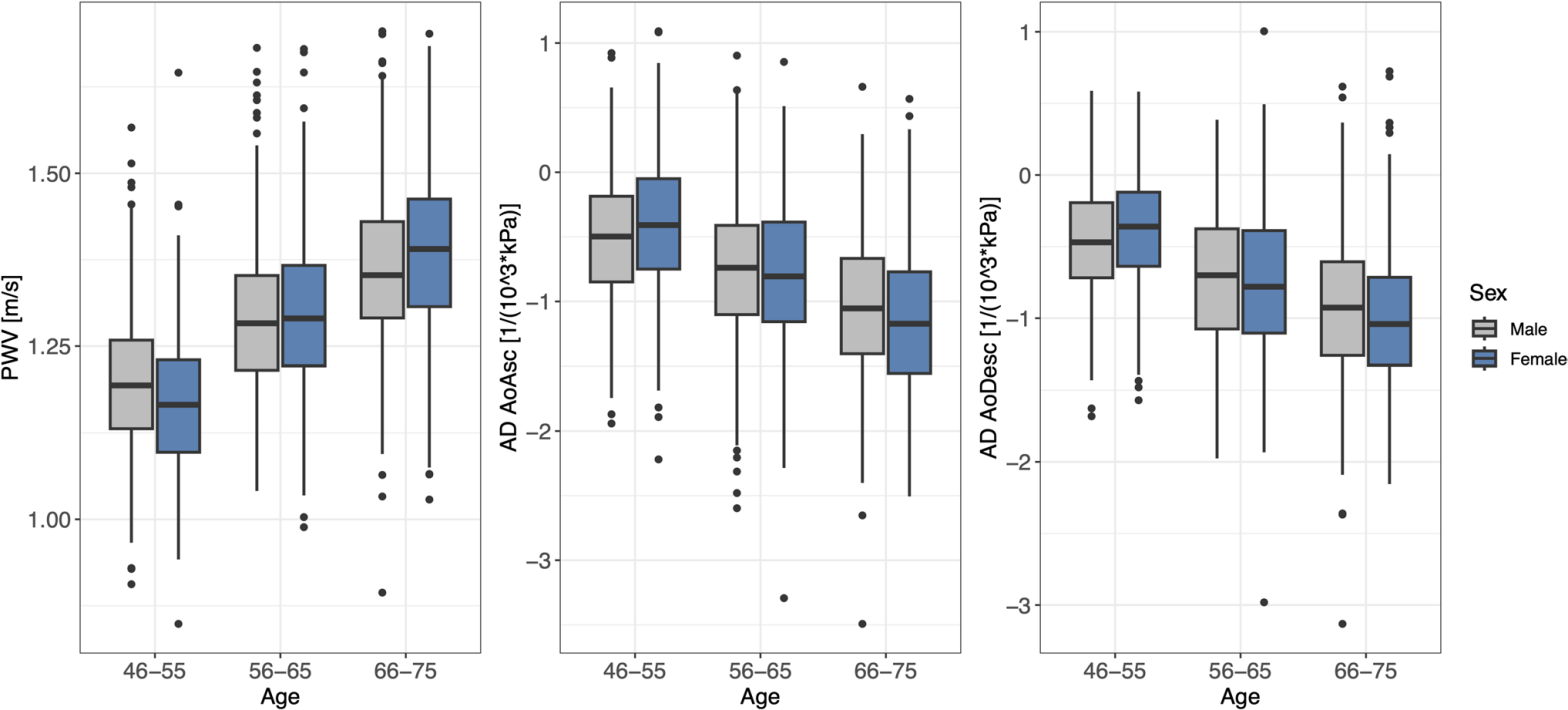
Boxplots of the Box-Cox transformed PWV (**a**), AD AoAsc (**b**), and AD AoDesc (**c**) according to age (PWV, AD AoAsc, and AD AoDesc: all *p* < 0.001) and to sex categories (PWV: *p* = 0.978, AD AoAsc: *p* = 0.247, AD AoDesc: *p* = 0.216). *PWV*, *pulse wave velocity*; *AD*, *aortic distensibility*;* AoAsc*, *ascending aorta*;* AoDesc*, *descending aorta*

Median PWV values increased with age (age 46–55: 5.54 [4.75, 6.51] m/s, age 56–64: 7.08 [6.02, 8.53] m/s, age 66–75: 8.87 [7.28, 11.00] m/s), whereas median AD AoAsc (age 46–55: 0.63 [0.44, 0.85] [1/(10^3*kPa)], age 56–64: 0.46 [0.31, 0.66] [1/(10^3*kPa)], age 66–75: 0.32 [0.22, 0.47] [1/(10^3*kPa)]) and AD AoDesc (age 46–55: 0.65 [0.49, 0.86] [1/(10^3*kPa)], age 56–64: 0.46 [0.31, 0.67] [1/(10^3*kPa)], age 66–75: 0.35 [0.24, 0.51] [1/(10^3*kPa)]) values decreased with age. PWV, AD AoAsc, and AD AoDesc correlated significantly with age (Pearson correlation: *r* = 0.564 [0.535, 0.592], *p* < 0.001; *r* = −0.435 [−0.468, −0.401], *p* < 0.001; *r = *−0.404 [−0.438, −0.369], *p* < 0.001) (Table 3, Fig. 3).

**Table 3:** Median PWV, AD AoAsc, and AD AoDesc according to age categories and sex

	Age groups (years)				Sex		
	46–55	56–65	66–75	***p***-value	Male	Female	***p***-value
PWV [m/s]	5.54 [4.75, 6.51]	7.08 [6.02, 8.53]	8.87 [7.28, 11.00]	< 0.001	7.60 [6.28, 9.60]	7.82 [6.03, 10.01]	0.978
AD AoAsc [1/(10^3*kPa)]	0.63 [0.44, 0.85]	0.46 [0.31, 0.66]	0.32 [0.22, 0.47]	< 0.001	0.40 [0.26, 0.60]	0.38 [0.24, 0.61]	0.247
AD AoDesc [1/(10^3*kPa)]	0.65 [0.49, 0.86]	0.46 [0.31, 0.67]	0.35 [0.24, 0.51]	< 0.001	0.44 [0.28, 0.65]	0.41 [0.26, 0.63]	0.216

*PWV*, pulse wave velocity; *AD*, aortic distensibility; *AoAsc*, ascending aorta; *AoDesc*, descending aorta

Median PWV value was higher in females compared to males (7.82 [6.28, 9.60] m/s vs. 7.60 [6.28, 9.60] m/s). AD AoAsc and AD AoDesc were lower in female participants than in male participants (0.38 [0.24, 0.61] [1/(10^3*kPa)] vs. 0.40 [0.26, 0.60] [1/(10^3*kPa)] and 0.41 [0.26, 0.63] [1/(10^3*kPa)] vs. 0.44 [0.28, 0.65] [1/(10^3*kPa)]). However, there were no significant associations with sex (*p* = 0.978, *p* = 0.247, *p* = 0.216) (Table 3, Fig. 3).

### Associations of PWV and AD with CVRF and/or CVD after adjustment for age and sex

**Fig. 4:**
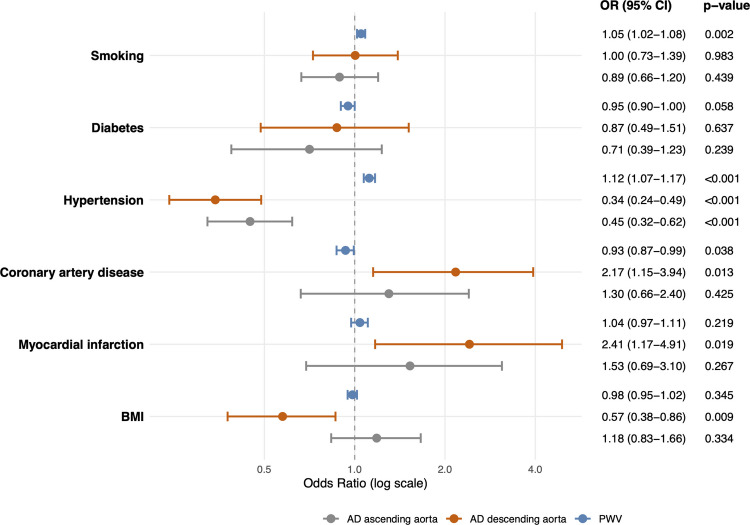
Forrest plot for associations of PWV, AD AoAsc, and AD AoDesc with CVRF and/or CVD after adjustment for age and sex. *PWV*, *pulse wave velocity*;* AD*, *aortic distensibility*;* AoAsc*, *ascending aorta*; *AoDesc,* *descending aorta*; *BMI*, *body mass index*;* CVRF*, *cardiovascular risk factors*;* CVD*, *cardiovascular diseases*

After adjustment for age and sex, higher PWV values were significantly associated with hypertension (OR 1.118 [1.072, 1.169], *p* < 0.001) and current or history of smoking (OR 1.050 [1.018, 1.084], *p* = 0.002), whereas lower PWV values were significantly associated with coronary artery disease (OR 0.932 [0.87, 0.993], *p* = 0.038). Lower AD AoAsc values were significantly associated only with hypertension (OR 0.448 [0.323, 0.619], *p* < 0.001). Lower AD AoDesc values were significantly associated with hypertension (OR 0.343 [0.241, 0.488], *p* < 0.001) and BMI (OR 0.575 [0.377, 0.863], *p* = 0.009). Higher AD AoDesc values were significantly associated with coronary artery disease (OR 2.17 [1.152, 3.936], *p* = 0.013) and with a history of myocardial infarction (OR 2.413 [1.117, 4.911], *p* = 0.019) (S1 Table, Fig. 4).

## Discussion

This study quantified CMR-based PWV and AD, evaluated their associations with CVRF and/or CVD, and discussed their role as a risk prediction factor in the large population-based Hamburg City Health Study. Median PWV was significantly higher, and median AD was significantly lower in the CVRF/CVD + group, respectively. After adjustment for age and sex, higher PWV but lower AD AoAsc and AD AoDesc values were significantly associated with hypertension. In contrast, lower PWV and higher AD AoDesc values were significantly associated with coronary artery disease and with coronary artery disease and/or a history of myocardial infarction, respectively (Graphical abstract).

### AS quantification in the HCHS cohort

Our large population-based study enables the analysis of CMR-AS data comparing the CVRF/CVD + group and the CVRF/CVD − group, representing cardiovascular “healthy” participants. Median PWV was 5.80 [4.91, 7.19] m/s in our “healthy” cohort. This finding is well in line with data by Harloff et al*.*, reference values by Kawel-Boehm et al*.*, and the NEO study [6, 21, 22]. Additionally, median AD of the ascending and the descending aorta were 0.54 and 0.61 [1/(10^3*kPa)] in the HCHS, which also confirm data of the UK Biobank and reference values by Kawel-Boehm et al*.* [15, 22]. Our analyses provide AS data in the cardiovascular “healthy” individuals, but for individuals with CVRF/CVD, who represent the vast majority of the general population [23–27], expanding previous knowledge in a large, population-based setting.

### Associations with age and sex

We found significant correlations of PWV and AD with age, but no significant association with sex **(**Table 3,Fig. 3**)**. A recent longitudinal, population-based study confirmed individually increasing PWV and decreasing AD values with ageing in females as well as in males [19]. Age is known to be the major determinant for PWV and AD reflecting the increasing stiffening of ageing vessels [5, 6, 21, 28]. This phenomenon can be explained by age-related inflammatory processes and oxidative stress, leading to fatigue fractures of elastic fibers [6, 29]. In our cohort, sex was not significantly associated with PWV and AD despite a non-significant tendency in older participants (Table 3, Fig. 3). These data are in line with the NEO study, which found increasing PWV values with ageing, but no significant differences between females and males [21]. In contrast, Harloff et al*.* analyzed 126 apparently healthy individuals and found PWV to increase and AD to decrease with age and to be lower in females compared to males [6]. In addition, the UK Biobank reported significant differences in AD between females and male participants [15]. The less pronounced sex-related differences of PWV and AD [5, 6, 21, 30] could be explained by the diminished influence of hormones on the aortic wall structure [6] in cohorts with older and mainly post-menopausal females, such as in our study population, compared to other studies with more pronounced sex-related differences [21]. Further studies are needed to address sex-specific differences in longitudinal changes of PWV and AD parameter with ageing between males and females, as well as possible effects of body size or blood pressure on sex effects.

### Associations with hypertension

Hypertension was the only CVRF to be significantly and consistently associated with all aortic stiffness parameters after adjustment for age and sex (S1 Table, Fig. 4). Our findings agree with the results from recent studies on this topic: Harloff et al*.*[6], the NEO CMR substudy [21] and the UK Biobank consistently demonstrated associations of aortic stiffness parameters with hypertension [12]. Hypertension is associated with aortic stiffness in several ways: Briefly, “stiffer” arteries result in a faster transit of the pulse wave, which can lead to higher blood pressure values, hereby promoting hypertension itself [12, 31]. However, it remains unclear so far whether aortic stiffness actually precedes hypertension, in terms of a pathophysiological factor, or is, vice versa, rather a sequela of hypertension [32]. Most likely, aortic stiffness and hypertension influence and aggravate each other as part of a vicious circle [32]. Independent from the exact pathophysiology, our findings underscore the key role of hypertension for aortic stiffness. Nevertheless, a causal relationship between hypertension and aortic stiffness cannot be inferred from our data due to the cross-sectional, population-based design of the Hamburg City Health Study. Further, in particular, longitudinal analyses are required to better address this issue. Current ESC guidelines for hypertension recommended PWV quantification for additional individual risk assessment with a IIb recommendation level, and our findings indicate a potential role of CMR measurements of AS, which could easily be integrated into standard CMR protocols as an adjunct feature [33].

### Associations with other CVRF

Associations of aortic function with CVRF can be interpreted as correlates of (early) atherosclerosis, but also by direct effects on vascular function [10, 34]. However, there are conflicting findings on this topic in current literature [35], possibly due to paradoxically decreased PWV values in the early stages of atherosclerosis [36]. We found a significant association of PWV with smoking, confirming recent data from the UK Biobank [12] (S1 Table, Fig. 4). This finding can be explained by smoking-induced vascular damage, related to inflammatory processes, endothelial dysfunction, and oxidative stress [37, 38], with subsequent changes in vascular tone and increased aortic stiffness [12, 39].

Obesity was associated with lower AD values of the descending aorta in our study. Harloff et al*.* found obesity to be associated with increased PWV values, but only 10% of the cohort was obese [6] and may not be representative to address this aspect adequately. Furthermore, current literature regarding obesity and aortic stiffness demonstrates controversial data [40–42]. This may be explained by differences in age distribution, obesity prevalence, and imaging methodology, indicating a need for further studies focusing on this topic.

### Associations with CVD

PWV and AD of the descending aorta were significantly associated with CAD after adjusting for age and sex (S1 Table, Fig. 4). However, the OR of PWV for CAD was < 1.0 and > 1.0 of AD for CAD, respectively (S1 Table, Fig. 4). These findings would suggest that “healthier” PWV and AD values were associated with CAD in this cross-sectional study cohort, apparently contradicting recent findings that higher PWV and lower AD values were associated with the presence [43], extent of CAD [44] and adverse prognosis [45, 46]. However, this counterintuitive finding could be related to the study design and population, including confounding factors such as treatment/behavioral effects due to the diagnosis of CAD, survivor bias after MI, or residual confounding: In particular, our analysis is a cross-sectional study, and PWV and AD values could have been confounded by medical treatment in participants with a history of CAD and/or MI. Moreover, participants with CAD and/or MI were significantly older compared to participants without a history of CAD and/or MI (*p* < 0.001) (S2 Table, S1 Fig.). Therefore, the strong effect of age on PWV and AD, but also on the risk for CAD and MI at the same time, could have confounded this analysis. Fully addressing this topic would require a large, longitudinal study and goes beyond the scope of our population-based study. In any case, our findings do not indicate a predictive value of abnormal PWV and AD values for prevalent CAD and MI.

### Limitations

General potential limitations of this study include the single-center design and the male-to-female ratio, which, similar to other studies on this topic, was not completely balanced [12, 45]. In the HCHS, the rate of participants in the CVRF/CVD + group was high at 92%, whereas the NEO study reported only a rate of 56% [21]. However, in the NEO study, a BMI ≥ 27 kg/m^2^ was an inclusion criterion for CMR scans compared with the HCHS, where BMI was defined as a CVRF [21], which could explain this difference. We investigated models with adjustments for the classical risk factors (BMI, hypertension, diabetes, smoking, lipids). However, these models were unstable (covariance matrix almost singular with a determinant of 1.9 E-21), so we have to refrain from reporting these more complex models. A potential technical limitation is the use of a 2D flow CMR method in this study, which constitutes a difference compared to recent studies using 4D flow measurements [6, 19]. However, a 2D flow CMR approach might be better suited for clinical CMR protocols due to fast data acquisition and analyses, especially in large population-based studies, and more likely to be implemented into clinical routine compared to time-consuming 4D flow scans and post-processing with similar reproducibility [47]. In the HCHS, blood pressure was measured before the CMR scan, whereas no standardized blood pressure measurement protocols for aortic stiffness quantification were available. However, we measured blood pressure after the preparation time of the participants as a relaxing time. Of note, AS is a dynamic parameter, which can be influenced by changes in medication and/or physical activity [48], but an analysis of these potential confounders is beyond the scope of this cross-sectional, population-based study.

## Conclusions

The presence of CVRF and/or CVD is related to higher PWV and significantly lower AD values. However, hypertension is the only CVRF/CVD consistently associated with higher PWV and lower AD after adjustment for age and sex. Our findings do not indicate a predictive value of abnormal PWV and AD values for prevalent CAD and MI.

## Supplementary Information

Below is the link to the electronic supplementary material.

ESM1 (DOCX 5.19 MB)

## References

[1] Smulyan H, Mookherjee S, Safar ME (2016) The two faces of hypertension: role of aortic stiffness. J Am Soc Hypertens 10:175–183. 10.1016/j.jash.2015.11.012 26725014 10.1016/j.jash.2015.11.012

[2] Chirinos JA, Segers P, Hughes T, Townsend R (2019) Large-artery stiffness in health and disease: JACC state-of-the-art review. J Am Coll Cardiol 74:1237–1263. 10.1016/j.jacc.2019.07.012 31466622 10.1016/j.jacc.2019.07.012 PMC6719727

[3] Ibrahim el SH, Johnson KR, Miller AB, Shaffer JM, White RD (2010) Measuring aortic pulse wave velocity using high-field cardiovascular magnetic resonance: comparison of techniques. J Cardiovasc Magn Reson 12:26. 10.1186/1532-429x-12-26 20459799 10.1186/1532-429X-12-26 PMC2874535

[4] Harloff A, Zech T, Frydrychowicz A, Schumacher M, Schöllhorn J, Hennig J, Weiller C, Markl M (2009) Carotid intima-media thickness and distensibility measured by MRI at 3 T versus high-resolution ultrasound. Eur Radiol 19:1470–1479. 10.1007/s00330-009-1295-8 19214524 10.1007/s00330-009-1295-8

[5] Voges I, Jerosch-Herold M, Hedderich J, Pardun E, Hart C, Gabbert DD, Hansen JH, Petko C, Kramer HH, Rickers C (2012) Normal values of aortic dimensions, distensibility, and pulse wave velocity in children and young adults: a cross-sectional study. J Cardiovasc Magn Reson 14:77. 10.1186/1532-429x-14-77 23151055 10.1186/1532-429X-14-77 PMC3514112

[6] Harloff A, Mirzaee H, Lodemann T, Hagenlocher P, Wehrum T, Stuplich J, Hennemuth A, Hennig J, Grundmann S, Vach W (2018) Determination of aortic stiffness using 4D flow cardiovascular magnetic resonance - a population-based study. J Cardiovasc Magn Reson 20:43. 10.1186/s12968-018-0461-z 29925388 10.1186/s12968-018-0461-z PMC6011486

[7] Segers P, Rietzschel ER, Chirinos JA (2020) How to measure arterial stiffness in humans. Arterioscler Thromb Vasc Biol 40:1034–1043. 10.1161/atvbaha.119.313132 31875700 10.1161/ATVBAHA.119.313132 PMC7180118

[8] Sethi S, Rivera O, Oliveros R, Chilton R (2014) Aortic stiffness: pathophysiology, clinical implications, and approach to treatment. Integr Blood Press Control 7:29–34. 10.2147/ibpc.S59535 24910511 10.2147/IBPC.S59535 PMC4046515

[9] Valencia-Hernández CA, Lindbohm JV, Shipley MJ, Wilkinson IB, McEniery CM, Ahmadi-Abhari S, Singh-Manoux A, Kivimäki M, Brunner EJ (2022) Aortic pulse wave velocity as adjunct risk marker for assessing cardiovascular disease risk: prospective study. Hypertension 79:836–843. 10.1161/hypertensionaha.121.17589 35139665 10.1161/HYPERTENSIONAHA.121.17589 PMC9148390

[10] Vlachopoulos C, Aznaouridis K, O’Rourke MF, Safar ME, Baou K, Stefanadis C (2010) Prediction of cardiovascular events and all-cause mortality with central haemodynamics: a systematic review and meta-analysis. Eur Heart J 31:1865–1871. 10.1093/eurheartj/ehq024 20197424 10.1093/eurheartj/ehq024

[11] Pereira T, Correia C, Cardoso J (2015) Novel methods for pulse wave velocity measurement. J Med Biol Eng 35:555–565. 10.1007/s40846-015-0086-8 26500469 10.1007/s40846-015-0086-8 PMC4609308

[12] Said MA, Eppinga RN, Lipsic E, van der Harst P (2018) Relationship of arterial stiffness index and pulse pressure with cardiovascular disease and mortality. J Am Heart Assoc 7:10.1161/jaha.117.007621 10.1161/JAHA.117.007621 PMC5850166 29358193

[13] Grotenhuis HB, Westenberg JJ, Steendijk P, van der Geest RJ, Ottenkamp J, Bax JJ, Jukema JW, de Roos A (2009) Validation and reproducibility of aortic pulse wave velocity as assessed with velocity-encoded MRI. J Magn Reson Imaging 30:521–526 19711407 10.1002/jmri.21886

[14] Laurent S, Cockcroft J, Van Bortel L, Boutouyrie P, Giannattasio C, Hayoz D, Pannier B, Vlachopoulos C, Wilkinson I, Struijker-Boudier H (2006) Expert consensus document on arterial stiffness: methodological issues and clinical applications. Eur Heart J 27:2588–2605. 10.1093/eurheartj/ehl254 17000623 10.1093/eurheartj/ehl254

[15] Cecelja M, Ruijsink B, Puyol-Antón E, Li Y, Godwin H, King AP, Razavi R, Chowienczyk P (2022) Aortic distensibility measured by automated analysis of magnetic resonance imaging predicts adverse cardiovascular events in UK Biobank. J Am Heart Assoc 11:e026361. 10.1161/jaha.122.026361 36444831 10.1161/JAHA.122.026361 PMC9851433

[16] Bohnen S, Avanesov M, Jagodzinski A, Schnabel RB, Zeller T, Karakas M, Schneider J, Tahir E, Cavus E, Spink C, Radunski UK, Ojeda F, Adam G, Blankenberg S, Lund GK, Muellerleile K (2018) Cardiovascular magnetic resonance imaging in the prospective, population-based, Hamburg City Health cohort study: objectives and design. J Cardiovasc Magn Reson 20:68. 10.1186/s12968-018-0490-7 30244673 10.1186/s12968-018-0490-7 PMC6151919

[17] Jagodzinski A, Johansen C, Koch-Gromus U, Aarabi G, Adam G, Anders S, Augustin M, der Kellen RB, Beikler T, Behrendt CA, Betz CS, Bokemeyer C, Borof K, Briken P, Busch CJ, Büchel C, Brassen S, Debus ES, Eggers L, Fiehler J, Gallinat J, Gellißen S, Gerloff C, Girdauskas E, Gosau M, Graefen M, Härter M, Harth V, Heidemann C, Heydecke G, Huber TB, Hussein Y, Kampf MO, von dem Knesebeck O, Konoppka A, König HH, Kromer R, Kubisch C, Kühn S, Loges S, Löwe B, Lund G, Meyer C, Nagel L, Nienhaus A, Pantel K, Petersen E, Püschel K, Reichenspurner H, Sauter G, Scherer M, Scherschel K, Schiffner U, Schnabel RB, Schulz H, Smeets R, Sokalskis V, Spitzer MS, Terschüren C, Thederan I, Thoma T, Thomalla G, Waschki B, Wegscheider K, Wenzel JP, Wiese S, Zyriax BC, Zeller T, Blankenberg S (2020) Rationale and design of the Hamburg City Health Study. Eur J Epidemiol 35:169–181. 10.1007/s10654-019-00577-4 31705407 10.1007/s10654-019-00577-4 PMC7125064

[18] Kramer CM, Barkhausen J, Bucciarelli-Ducci C, Flamm SD, Kim RJ, Nagel E (2020) Standardized cardiovascular magnetic resonance imaging (CMR) protocols: 2020 update. J Cardiovasc Magn Reson 22:17. 10.1186/s12968-020-00607-1 32089132 10.1186/s12968-020-00607-1 PMC7038611

[19] Loose S, Solou D, Strecker C, Hennemuth A, Hüllebrand M, Grundmann S, Asmussen A, Treppner M, Urbach H, Harloff A (2023) Characterization of aortic aging using 3D multi-parametric MRI-long-term follow-up in a population study. Sci Rep 13:6285. 10.1038/s41598-023-33219-7 37072440 10.1038/s41598-023-33219-7 PMC10111081

[20] Wentland AL, Wieben O, François CJ, Boncyk C, Munoz Del Rio A, Johnson KM, Grist TM, Frydrychowicz A (2013) Aortic pulse wave velocity measurements with undersampled 4D flow-sensitive MRI: comparison with 2D and algorithm determination. J Magn Reson Imaging 37:853–859. 10.1002/jmri.23877 23124585 10.1002/jmri.23877 PMC3566322

[21] van Hout MJ, Dekkers IA, Westenberg JJ, Schalij MJ, Widya RL, de Mutsert R, Rosendaal FR, de Roos A, Jukema JW, Scholte AJ, Lamb HJ (2021) Normal and reference values for cardiovascular magnetic resonance-based pulse wave velocity in the middle-aged general population. J Cardiovasc Magn Reson 23:46. 10.1186/s12968-021-00739-y 33866975 10.1186/s12968-021-00739-y PMC8054386

[22] Kawel-Boehm N, Hetzel SJ, Ambale-Venkatesh B, Captur G, Chin CWL, François CJ, Jerosch-Herold M, Luu JM, Raisi-Estabragh Z, Starekova J, Taylor M, van Hout M, Bluemke DA (2025) Society for Cardiovascular Magnetic Resonance reference values (“normal values”) in cardiovascular magnetic resonance: 2025 update. J Cardiovasc Magn Reson 27:10.1016/j.jocmr.2025.101853 10.1016/j.jocmr.2025.101853 PMC12159681 39914499

[23] Wolf-Maier K, Cooper RS, Banegas JR, Giampaoli S, Hense HW, Joffres M, Kastarinen M, Poulter N, Primatesta P, Rodríguez-Artalejo F, Stegmayr B, Thamm M, Tuomilehto J, Vanuzzo D, Vescio F (2003) Hypertension prevalence and blood pressure levels in 6 European countries, Canada, and the United States. JAMA 289:2363–2369. 10.1001/jama.289.18.2363 12746359 10.1001/jama.289.18.2363

[24] Tamayo T, Brinks R, Hoyer A, Kuß OS, Rathmann W (2016) The prevalence and incidence of diabetes in Germany. Dtsch Arztebl Int 113:177–182. 10.3238/arztebl.2016.0177 27118665 10.3238/arztebl.2016.0177 PMC4850517

[25] Gößwald A, Schienkiewitz A, Nowossadeck E, Busch MA (2013) Prevalence of myocardial infarction and coronary heart disease in adults aged 40–79 years in Germany: results of the German Health Interview and Examination Survey for Adults (DEGS1). Bundesgesundheitsbl Gesundheitsforsch Gesundheitsschutz 56:650–655. 10.1007/s00103-013-1666-9 10.1007/s00103-013-1666-9 23703482

[26] Zeiher J, Kuntz B, Lange C (2017) Smoking among adults in Germany. J Health Monit 2:57–63. 10.17886/rki-gbe-2017-043 37152097 10.17886/RKI-GBE-2017-043 PMC10161277

[27] Ballena-Caicedo J, Zuzunaga-Montoya FE, Loayza-Castro JA, Vásquez-Romero LEM, Tapia-Limonchi R, De Carrillo CIG, Vera-Ponce VJ (2025) Global prevalence of dyslipidemias in the general adult population: a systematic review and meta-analysis. J Health Popul Nutr 44:308. 10.1186/s41043-025-01054-3 40859400 10.1186/s41043-025-01054-3 PMC12379389

[28] Westenberg JJ, Scholte AJ, Vaskova Z, van der Geest RJ, Groenink M, Labadie G, van den Boogaard PJ, Radonic T, Hilhorst-Hofstee Y, Mulder BJ, Kroft LJ, Reiber JH, de Roos A (2011) Age-related and regional changes of aortic stiffness in the Marfan syndrome: assessment with velocity-encoded MRI. J Magn Reson Imaging 34:526–531. 10.1002/jmri.22646 21761466 10.1002/jmri.22646

[29] Donato AJ, Machin DR, Lesniewski LA (2018) Mechanisms of dysfunction in the aging vasculature and role in age-related disease. Circ Res 123:825–848. 10.1161/circresaha.118.312563 30355078 10.1161/CIRCRESAHA.118.312563 PMC6207260

[30] Kim EK, Chang SA, Jang SY, Kim Y, Kim SM, Oh JK, Choe YH, Kim DK (2013) Assessment of regional aortic stiffness with cardiac magnetic resonance imaging in a healthy Asian population. Int J Cardiovasc Imaging 29(1):57–64. 10.1007/s10554-013-0206-x 23504214 10.1007/s10554-013-0206-x

[31] Westerhof N, O’Rourke MF (1995) Haemodynamic basis for the development of left ventricular failure in systolic hypertension and for its logical therapy. J Hypertens 13:943–952. 10.1097/00004872-199509000-00002 8586828 10.1097/00004872-199509000-00002

[32] Humphrey JD, Harrison DG, Figueroa CA, Lacolley P, Laurent S (2016) Central artery stiffness in hypertension and aging: a problem with cause and consequence. Circ Res 118:379–381. 10.1161/circresaha.115.307722 26846637 10.1161/CIRCRESAHA.115.307722 PMC4745997

[33] McEvoy JW, McCarthy CP, Bruno RM, Brouwers S, Canavan MD, Ceconi C, Christodorescu RM, Daskalopoulou SS, Ferro CJ, Gerdts E, Hanssen H, Harris J, Lauder L, McManus RJ, Molloy GJ, Rahimi K, Regitz-Zagrosek V, Rossi GP, Sandset EC, Scheenaerts B, Staessen JA, Uchmanowicz I, Volterrani M, Touyz RM, Group ESD (2024) 2024 ESC Guidelines for the management of elevated blood pressure and hypertension: developed by the task force on the management of elevated blood pressure and hypertension of the European Society of Cardiology (ESC) and endorsed by the European Society of Endocrinology (ESE) and the European Stroke Organisation (ESO). Eur Heart J. 10.1093/eurheartj/ehae178

[34] Cavalcante JL, Lima JA, Redheuil A, Al-Mallah MH (2011) Aortic stiffness: current understanding and future directions. J Am Coll Cardiol 57:1511–1522. 10.1016/j.jacc.2010.12.017 21453829 10.1016/j.jacc.2010.12.017

[35] Cecelja M, Chowienczyk P (2009) Dissociation of aortic pulse wave velocity with risk factors for cardiovascular disease other than hypertension: a systematic review. Hypertension 54:1328–1336. 10.1161/hypertensionaha.109.137653 19884567 10.1161/HYPERTENSIONAHA.109.137653

[36] Farrar DJ, Bond MG, Riley WA, Sawyer JK (1991) Anatomic correlates of aortic pulse wave velocity and carotid artery elasticity during atherosclerosis progression and regression in monkeys. Circulation 83:1754–1763. 10.1161/01.cir.83.5.1754 2022028 10.1161/01.cir.83.5.1754

[37] Cacciola RR, Guarino F, Polosa R (2007) Relevance of endothelial-haemostatic dysfunction in cigarette smoking. Curr Med Chem 14:1887–1892. 10.2174/092986707781058832 17627524 10.2174/092986707781058832

[38] Esen AM, Barutcu I, Acar M, Degirmenci B, Kaya D, Turkmen M, Melek M, Onrat E, Esen OB, Kirma C (2004) Effect of smoking on endothelial function and wall thickness of brachial artery. Circ J 68:1123–1126. 10.1253/circj.68.1123 15564694 10.1253/circj.68.1123

[39] Binder S, Navratil K, Halek J (2008) Chronic smoking and its effect on arterial stiffness. Biomed Pap Med Fac Univ Palacky Olomouc Czech Repub 152:299–302. 10.5507/bp.2008.047 19219224 10.5507/bp.2008.047

[40] Kim H-L, Ahn D-W, Kim SH, Lee DS, Yoon SH, Zo J-H, Kim M-A, Jeong JB (2021) Association between body fat parameters and arterial stiffness. Sci Rep 11:20536. 10.1038/s41598-021-00175-z 34654852 10.1038/s41598-021-00175-z PMC8519992

[41] Rider OJ, Tayal U, Francis JM, Ali MK, Robinson MR, Byrne JP, Clarke K, Neubauer S (2010) The effect of obesity and weight loss on aortic pulse wave velocity as assessed by magnetic resonance imaging. Obesity Silver Spring 18:2311–2316. 10.1038/oby.2010.64 20360756 10.1038/oby.2010.64

[42] Wildman RP, Mackey RH, Bostom A, Thompson T, Sutton-Tyrrell K (2003) Measures of obesity are associated with vascular stiffness in young and older adults. Hypertension 42:468–473. 10.1161/01.Hyp.0000090360.78539.Cd 12953016 10.1161/01.HYP.0000090360.78539.CD

[43] Lever-Megina CG, Cavero-Redondo I, Álvarez-Bueno C, Morales-Berenkova C, Cabeza-Arrebola G, Saz-Lara A (2025) Accuracy of pulse wave velocity for screening coronary artery disease: a systematic review and meta-analysis. Diagnosis Berl. 10.1515/dx-2024-0193 39814690 10.1515/dx-2024-0193

[44] Ejiri K, Ding N, Kim E, Honda Y, Cainzos-Achirica M, Tanaka H, Howard-Claudio CM, Butler KR, Hughes TM, Van’t Hof JR, Meyer ML, Blaha MJ, Matsushita K (2024) Association of segment-specific pulse wave velocity with vascular calcification: the ARIC (atherosclerosis risk in communities) study. J Am Heart Assoc 13:e031778. 10.1161/jaha.123.031778 38214278 10.1161/JAHA.123.031778 PMC10926832

[45] Maroules CD, Khera A, Ayers C, Goel A, Peshock RM, Abbara S, King KS (2014) Cardiovascular outcome associations among cardiovascular magnetic resonance measures of arterial stiffness: the Dallas heart study. J Cardiovasc Magn Reson 16:33. 10.1186/1532-429x-16-33 24886531 10.1186/1532-429X-16-33 PMC4031496

[46] Chen C, Bao W, Chen C, Chen L, Wang L, Gong H (2023) Association between estimated pulse wave velocity and all-cause mortality in patients with coronary artery disease: a cohort study from NHANES 2005–2008. BMC Cardiovasc Disord 23:412. 10.1186/s12872-023-03435-0 37605157 10.1186/s12872-023-03435-0 PMC10441734

[47] Markl M, Wallis W, Brendecke S, Simon J, Frydrychowicz A, Harloff A (2010) Estimation of global aortic pulse wave velocity by flow-sensitive 4D MRI. Magn Reson Med 63:1575–1582. 10.1002/mrm.22353 20512861 10.1002/mrm.22353

[48] Deiseroth A, Streese L, Köchli S, Wüst RS, Infanger D, Schmidt-Trucksäss A, Hanssen H (2019) Exercise and arterial stiffness in the elderly: a combined cross-sectional and randomized controlled trial (EXAMIN AGE). Front Physiol 10:1119. 10.3389/fphys.2019.01119 31551805 10.3389/fphys.2019.01119 PMC6738015

