# Supplementary material for: Associations between cardiovascular risk factors and diseases with aortic pulse wave velocity and aortic distensibility: magnetic resonance imaging in the Hamburg city health study
Source: Clin Res Cardiol. 2026 Feb 9;115(8):1389–99. doi: 10.1007/s00392-026-02866-x (PMC13346220; doi:10.1007/s00392-026-02866-x)
Supplement: Supplementary file 1 — (DOCX 5.19 MB) [file 392_2026_2866_MOESM1_ESM.docx]

**Supplemental Material**

**
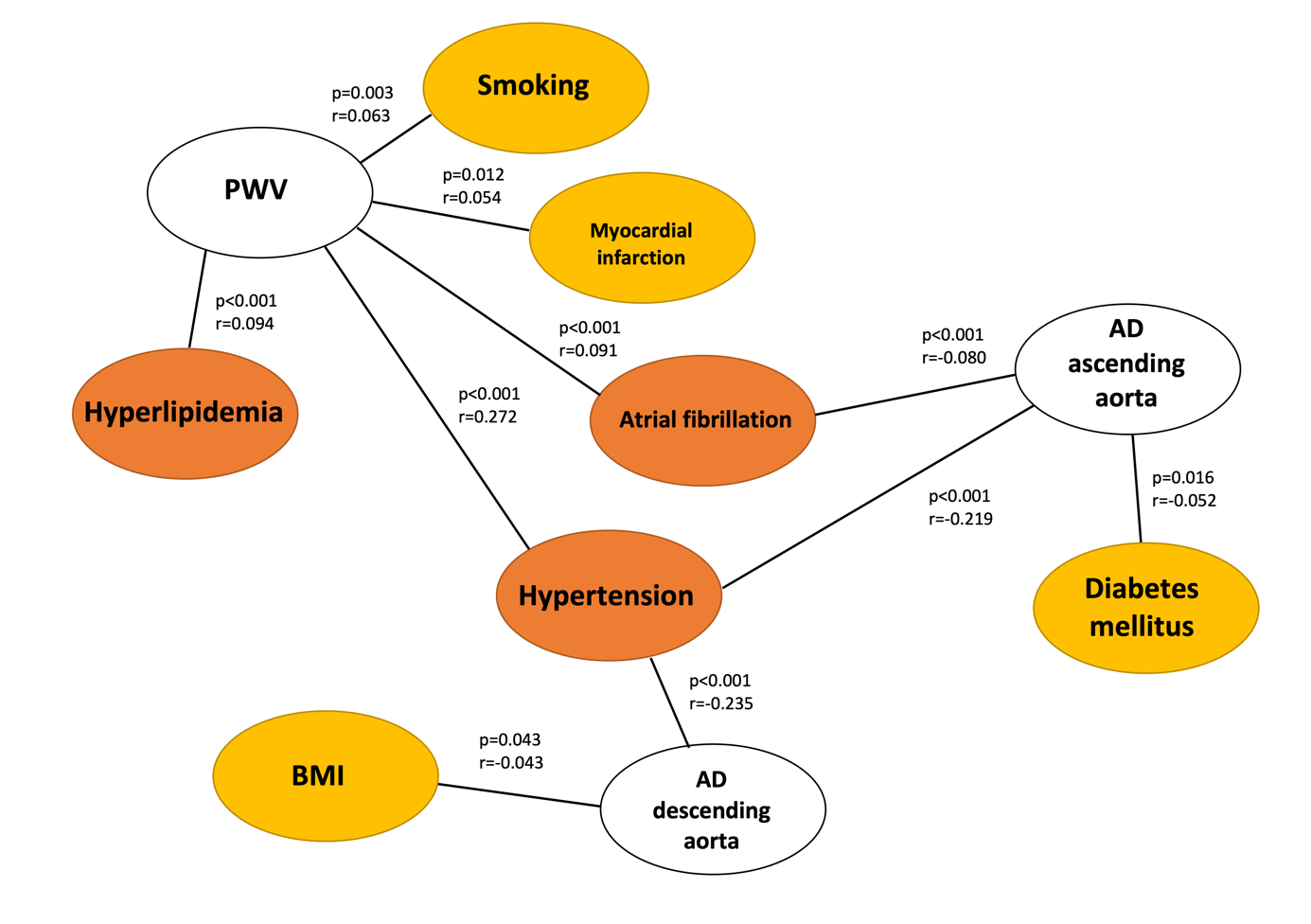
**

**S1 Fig. Regression Analysis for PWV 50% and AD according to Harloff *et al.* with CVRF and/or CVD without adjustment for age and sex**

*PWV = pulse wave velocity, AD = aortic distensibility, BMI = body mass index, CVRF = cardiovascular risk factors, CVD = cardiovascular diseases.*

**S1 Table Regression Analysis for PWV and AD with cardiovascular risk factors and/or diseases.**

1. **BMI**

|  | **PWV model** | | **AD AoDesc model** | | **AD AoAsc model** | |
| --- | --- | --- | --- | --- | --- | --- |
|  | **Odds ratio [95% CI]** | **p-value** | **Odds ratio [95% CI]** | **p-value** | **Odds ratio [95% CI]** | **p-value** |
| **PWV/AD variable** | 0.983 [0.947, 1.018] | 0.345 | 0.575 [0.377, 0.863] | 0.009 | 1.184 [0.835,  1.660] | 0.334 |
| **Age (years)** | 0.996 [0.982,  1.010] | 0.556 | 0.984 [0.971, 0.998] | 0.024 | 0.995 [0.981, 1.009] | 0.459 |
| **Sex (females)** | 1.029 [0.832, 1.271] | 0.789 | 1.01  [0.817, 1.246] | 0.927 | 1.015 [0.821, 1.252] | 0.891 |

1. **Diabetes mellitus**

|  | **PWV model** | | **AD AoDesc model** | | **AD AoAsc model** | |
| --- | --- | --- | --- | --- | --- | --- |
|  | **Odds ratio [95% CI]** | **p-value** | **Odds ratio [95% CI]** | **p-value** | **Odds ratio [95% CI]** | **p-value** |
| **PWV/AD variable** | 0.950 [0.899,  1.000] | 0.058 | 0.872 [0.486, 1.514] | 0.637 | 0.707 [0.388, 1.231] | 0.239 |
| **Age (years)** | 1.05  [1.028, 1.073] | < 0.001 | 1.038 [1.018, 1.06] | < 0.001 | 1.035 [1.014, 1.057] | 0.001 |
| **Sex (females)** | 0.736 [0.539, 0.996] | 0.05 | 0.748 [0.551, 1.008] | 0.06 | 0.747  [0.550,  1.006] | 0.058 |

1. **Hyperlipidemia**

|  | **PWV model** | | **AD AoDesc model** | | **AD AoAsc model** | |
| --- | --- | --- | --- | --- | --- | --- |
|  | **Odds ratio [95% CI]** | **p-value** | **Odds ratio [95% CI]** | **p-value** | **Odds ratio [95% CI]** | **p-value** |
| **PWV/AD variable** | 1.004 [0.973, 1.036] | 0.791 | 1.198 [0.827, 1.722] | 0.334 | 1.193 [0.839,  1.680] | 0.318 |
| **Age (years)** | 1.047 [1.033, 1.062] | < 0.001 | 1.051 [1.037, 1.066] | < 0.001 | 1.052 [1.037, 1.067] | < 0.001 |
| **Sex (females)** | 0.539 [0.438, 0.662] | < 0.001 | 0.552  [0.450,  0.676] | < 0.001 | 0.551 [0.449, 0.675] | < 0.001 |

1. **Smoking**

|  | **PWV model** | | **AD AoDesc model** | | **AD AoAsc model** | |
| --- | --- | --- | --- | --- | --- | --- |
|  | **Odds ratio [95% CI]** | **p-value** | **Odds ratio [95% CI]** | **p-value** | **Odds ratio [95% CI]** | **p-value** |
| **PWV/AD variable** | 1.05  [1.018, 1.084] | 0.002 | 1.004 [0.726, 1.392] | 0.983 | 0.89  [0.664, 1.197] | 0.439 |
| **Age (years)** | 0.993 [0.981, 1.005] | 0.256 | 1.002  [0.99, 1.013] | 0.777 | 1  [0.988, 1.011] | 0.955 |
| **Sex (females)** | 0.607 [0.508, 0.724] | < 0.001 | 0.614 [0.515, 0.731] | < 0.001 | 0.613 [0.515, 0.731] | < 0.001 |

1. **Atrial Fibrillation**

|  | **PWV model** | | **AD AoDesc model** | | **AD AoAsc model** | |
| --- | --- | --- | --- | --- | --- | --- |
|  | **Odds ratio [95% CI]** | **p-value** | **Odds ratio [95% CI]** | **p-value** | **Odds ratio [95% CI]** | **p-value** |
| **PWV/AD variable** | 1.037 [0.975, 1.097] | 0.225 | 1.497 [0.657, 3.171] | 0.314 | 0.479 [0.168, 1.215] | 0.144 |
| **Age (years)** | 1.097 [1.059, 1.139] | < 0.001 | 1.113 [1.076, 1.155] | < 0.001 | 1.097  [1.060,  1.140] | < 0.001 |
| **Sex (females)** | 0.771 [0.489, 1.195] | 0.252 | 0.813 [0.521, 1.247] | 0.349 | 0.787 [0.505, 1.207] | 0.280 |

1. **Coronary artery disease**

|  | **PWV model** | | **AD AoDesc model** | | **AD AoAsc model** | |
| --- | --- | --- | --- | --- | --- | --- |
|  | **Odds ratio [95% CI]** | **p-value** | **Odds ratio [95% CI]** | **p-value** | **Odds ratio [95% CI]** | **p-value** |
| **PWV/AD variable** | 0.932  [0.870, 0.993] | 0.038 | 2.170  [1.152, 3.936] | 0.013 | 1.300  [0.661, 2.401] | 0.425 |
| **Age (years)** | 1.086 [1.057, 1.118] | < 0.001 | 1.081 [1.053, 1.111] | < 0.001 | 1.075 [1.046, 1.106] | < 0.001 |
| **Sex (females)** | 0.446 [0.292, 0.665] | < 0.001 | 0.446 [0.293, 0.660] | < 0.001 | 0.439 [0.289, 0.65] | < 0.001 |

1. **Hypertension**

|  | **PWV model** | | **AD AoDesc. model** | | **AD AoAsc model** | |
| --- | --- | --- | --- | --- | --- | --- |
|  | **Odds ratio [95% CI]** | **p-value** | **Odds ratio [95% CI]** | **p-value** | **Odds ratio [95% CI]** | **p-value** |
| **PWV/AD variable** | 1.118 [1.072, 1.169] | < 0.001 | 0.343 [0.241, 0.488] | < 0.001 | 0.448 [0.323, 0.619] | < 0.001 |
| **Age (years)** | 1.055 [1.041, 1.070] | < 0.001 | 1.060  [1.046, 1.073] | < 0.001 | 1.061 [1.048, 1.075] | < 0.001 |
| **Sex (females)** | 0.668 [0.546, 0.818] | < 0.001 | 0.683 [0.559, 0.835] | < 0.001 | 0.685 [0.561, 0.837] | < 0.001 |

1. **Myocardial infarction**

|  | **PWV model** | | **AD AoDesc model** | | **AD AoAsc model** | |
| --- | --- | --- | --- | --- | --- | --- |
|  | **Odds ratio [95% CI]** | **p-value** | **Odds ratio [95% CI]** | **p-value** | **Odds ratio [95% CI]** | **p-value** |
| **PWV/AD variable** | 1.041 [0.973, 1.106] | 0.219 | 2.413 [1.117, 4.911] | 0.019 | 1.529 [0.689, 3.096] | 0.267 |
| **Age (years)** | 1.048 [1.014, 1.085] | 0.006 | 1.063  [1.030, 1.098] | < 0.001 | 1.058 [1.025, 1.094] | 0.001 |
| **Sex (females)** | 0.305 [0.166, 0.523] | < 0.001 | 0.351 [0.198, 0.588] | < 0.001 | 0.346 [0.196, 0.580] | < 0.001 |

*PWV = pulse wave velocity, AD = aortic distensibility, BMI = body mass index, AoAsc = ascending aorta, AoDesc = descending aorta. Odds ratios are per unit change. For PWV the unit is m/s, for AD it is 1/(10^3*kPa).*

**S2 Table Comparison between participants without coronary artery disease and myocardial infarction and participants without coronary artery disease and/or myocardial infarction**

|  | **No coronary artery disease AND myocardial infarction**  (n=2067) | **Coronary artery disease AND/OR myocardial infarction**  **(**n=171) | ***p-value*** |
| --- | --- | --- | --- |
| **Age, years** | 66.00 [58.00, 71.00] | 70.00 [65.50, 73.00] | <0.001 |
| **Sex (females)** | 885 (42.82) | 41 (23.98) | <0.001 |
| **Systolic blood pressure before CMR [mmHg]** | 130.00 [120.00, 144.00] | 131.50 [116.25, 144.00] | 0.596 |
| **Diastolic blood pressure before CMR [mmHg]** | 79.00 [73.00, 86.00] | 77.00 [71.00, 84.00] | 0.007 |
| **Diabetes mellitus** | 176 (9.05) | 27 (15.79) | 0.006 |
| **Hypertension** | 1438 (71.97) | 159 (94.08) | <0.001 |
| **Hyperlipidemia** | 457 (23.51) | 126 (73.68) | <0.001 |
| **Smoking** | 1323 (64.07) | 128 (74.85) | 0.006 |
| **BMI (>30 kg/m^2^)** | 402 (20.14) | 41 (24.70) | 0.194 |
| **Stroke** | 56 (2.72) | 10 (5.85) | 0.037 |
| **Atrial fibrillation** | 77 (3.74) | 14 (8.48) | 0.006 |
| **Creatinine [mg/dl]** | 0.80 [0.73, 0.89] | 0.84 [0.76, 0.98] | <0.001 |
| **PWV [m/s]** | 7.63 [6.17, 9.81] | 8.17 [6.52, 9.93] | 0.039 |
| **AD AoAsc [1/(10^3*kPa)]** | 0.39 [0.26, 0.60] | 0.38 [0.24, 0.58] | 0.229 |
| **AD AoDesc [1/(10^3*kPa)]** | 0.42 [0.27, 0.64] | 0.44 [0.26, 0.65] | 0.686 |

*CMR = cardiovascular magnetic resonance, BMI = body mass index, PWV = pulse wave velocity, AD = aortic distensibility, AoAsc = ascending aorta, AoDesc = descending aorta.*
